# How do victims of bullying develop depression? Testing interpersonal style to explain the victimization‐depression link

**DOI:** 10.1111/jora.13005

**Published:** 2024-07-23

**Authors:** Minita Franzen, Marijtje A. J. van Duijn, Peter J. de Jong, René Veenstra, Marije aan het Rot

**Affiliations:** ^1^ Department of Psychology, Education & Child Studies, Erasmus School of Social and Behavioural Sciences Erasmus University Rotterdam Rotterdam The Netherlands; ^2^ Department of Psychology University of Groningen Groningen The Netherlands; ^3^ Department of Sociology University of Groningen Groningen The Netherlands

**Keywords:** assertiveness, depression, hostility, interpersonal functioning, victims of bullying

## Abstract

This study tested to what extent the relation between bullying victimization and future symptoms of depression could be explained by victims being more hostile and less assertive than non‐involved individuals. Data came from waves 2–4 of the Dutch TRacking Adolescents' Individual Lives Survey (TRAILS). Participants' bullying experiences were assessed at age 13, interpersonal style at age 16, and depression symptoms at age 19. Mediation analyses were performed primarily on 274 self‐reported victims and 1498 non‐involved peers. Self‐reported victims had an increased risk for depression symptoms. About a third of that risk could be explained by victims' hostile style, which was also higher than those of non‐involved peers. Although victims also reported lower levels of assertiveness than non‐involved peers, this interpersonal style did not mediate the link between bullying victimization and depression. Our findings suggest that high hostility, but not low assertiveness, partly explains the increased depression risk of self‐reported victims. Therefore, interventions could focus on addressing hostility, to help reduce the likelihood that adolescents who have experienced bullying victimization will have more interpersonal conflicts and mental health problems in the future. Supplementary materials also include analyses for bullies and bully‐victims, and for peer‐reported measures.

## INTRODUCTION

Bullying, whether traditional face‐to‐face or cyberbullying, can have severe consequences for all individuals involved. Focusing on internalizing problems and specifically depression, individuals who bully others (i.e., bullies), individuals who are bullied (i.e., victims), as well as individuals who both bully and are bullied (i.e., bully‐victims) have been reported to have more depression symptoms than individuals not involved in bullying, with bully‐victims having more symptoms than “pure” victims and both victim groups having more symptoms than “pure” bullies (e.g., Özdemir & Stattin, 2011; Swearer et al., 2001). There are also dose effects, with more frequent and intense victimization being associated with a greater risk for depression (e.g., van der Ploeg et al., 2015). Several past studies have found that bullying can predict later depression (Hemphill et al., 2015; Moore et al., 2017; Reijntjes et al., 2010; Ttofi et al., 2011). However, there is also evidence for depression being a predictor of later bullying experiences (Arseneault et al., 2010; Rudolph et al., 2008), suggesting a bi‐directional relation.

In line with most previous studies (Moore et al., 2017; Reijntjes et al., 2010; Ttofi et al., 2011), our starting point here is bullying predicting depression. We follow the call of various researchers to examine mediators of the victimization–depression relation, specifically the mediating role of interpersonal factors (Arseneault, 2018; Klomek et al., 2015). This is based on the increased recognition that it can be useful to examine bullying from a social–ecological perspective (Swearer & Espelage, 2004) and is also in line with the ecological–transactional model (Zimmer‐Gembeck, 2016). This perspective holds that in order to understand victimization and its antecedents and consequences, it is important to consider victims' interactions with others in their social environment (both offline and online), while also taking into account victims' interpersonal style and coping behaviors (Hymel & Swearer, 2015; Rodkin et al., 2015; Zimmer‐Gembeck, 2016).

### Interpersonal style of victims

In line with this social–ecological perspective, research has found that victims of both traditional and cyberbullying struggle interpersonally. For example, compared to their non‐involved peers, victims have been reported to lack assertiveness and behave submissively (Perren & Alsaker, 2006; Titova et al., 2022) and to react aggressively when triggered (Ferguson et al., 2016; Salmivalli & Helteenvuori, 2007; Salmivalli & Nieminen, 2002; Waasdorp & Bradshaw, 2011; Wright & Li, 2013). Overall, victims may tend to behave this way as a means of self‐protection from future bullying. However, according to the concept of interpersonal complementarity (Orford, 1986), submissiveness (i.e., non‐assertiveness) in one individual is usually responded to with dominance (i.e., assertiveness) in the interaction partner and vice versa, whereas a person's hostility usually invites hostility (also see Dawood et al., 2018). Therefore, victims' non‐assertive and hostile reactions can make them easy targets for (re)victimization by dominant, aggressive bullies (Salmivalli, 2010, 2014).

Importantly, these findings point toward victims having interpersonal styles that might generally be perceived as unpleasant, and that could increase the risk for future interpersonal struggles with others more generally (cf. Moskowitz, 2009, 2010). Victims' interpersonal styles might therefore also impact the quality of their social relationships overall, which means they fall short on this protective factor of subsequent mental health problems (cf. Ttofi et al., 2014).

### Interpersonal style and depression

**FIGURE 1:**
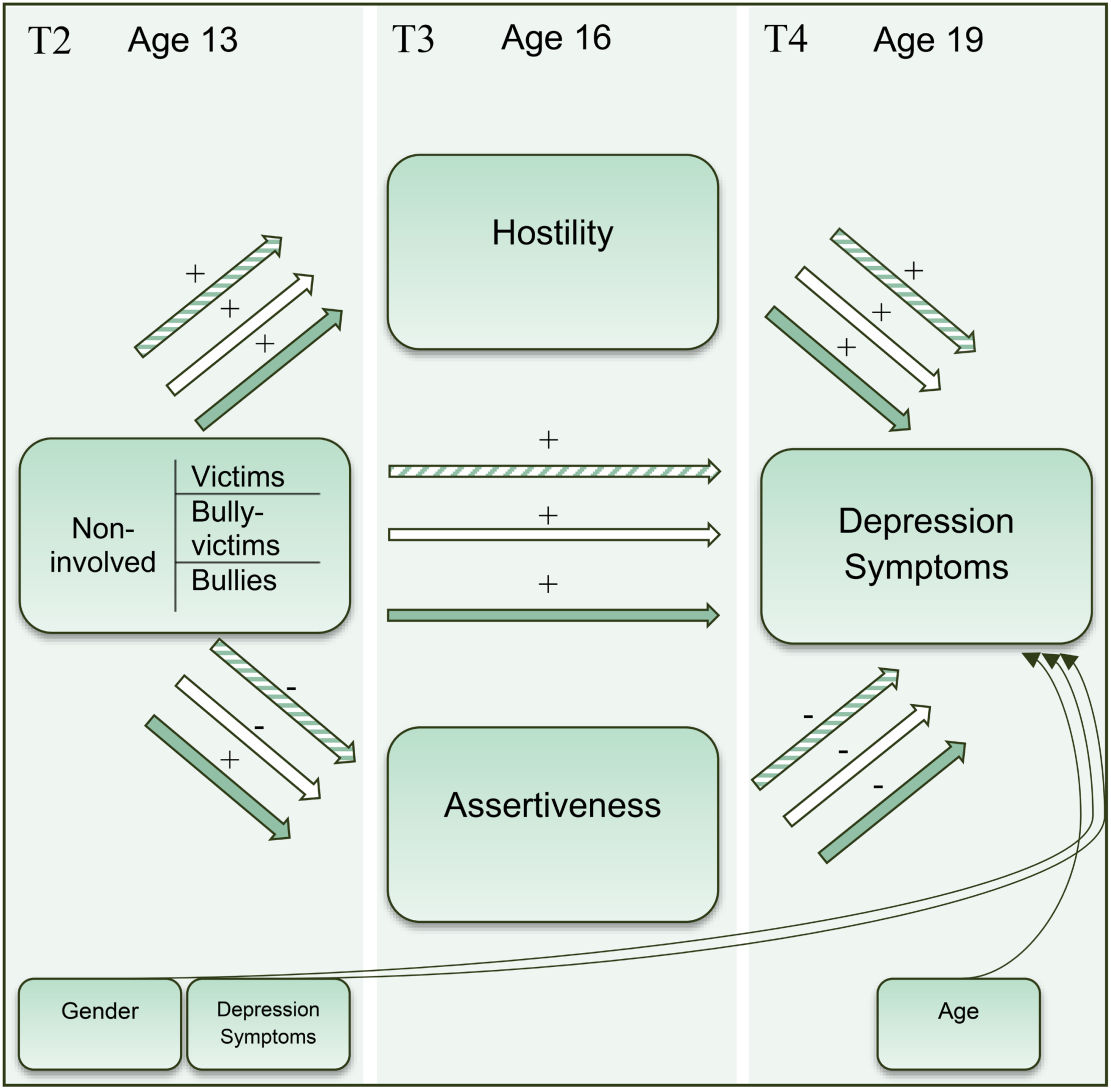
Illustration of mediation model. The model is based on dummy‐coded bullying victimization variables, with the non‐involved being the comparison group. Therefore, the striped arrows and respective signs represent the expected direction of the effect for the victim group as compared with the non‐involved group. The white arrow represents the expected effects for the bully‐victim dummy and the green arrow for the bully‐dummy.

Chronic interpersonal struggles and a lack of a stable support system have indeed been found to be unique risk factors for depression (Sato & McCann, 2007; Vrshek‐Schallhorn et al., 2015). More specifically, there are studies reporting non‐assertiveness/submissiveness in individuals with depression (e.g., Dawood et al., 2013). In addition, some studies report individuals with depression to have high levels of hostility (e.g., Dawood et al., 2013), though these associations are not always found (e.g., Girard et al., 2017). These findings point to the potential explanatory role of interpersonal processes in how individuals with a history of bullying victimization develop depression (see Figure 1).

### Interpersonal style as mediator of the victimization–depression relation

Multiple interpersonal factors (e.g., rejection sensitivity and emotion regulation) have already been tested as mediators of the association between victimization and internalizing mental health problems, including depression. However, most findings are based on cross‐sectional designs and small samples (for a review, see Kretschmer, 2016), or on very short‐term longitudinal designs of 3–6 months (Long et al., 2020; Noret et al., 2021). In the present study, we used data from a large, longitudinal, population‐based study on the psychological, social, and physical development of adolescents: the TRacking Adolescents' Individual Lives Survey (TRAILS). Covering a time span of 6 years (Waves 2–4, not including Wave 1, see Methods), this enabled us to examine assertiveness and hostility in a relatively substantial sample of victims, and whether these traits can explain their increased depression symptoms in comparison to non‐involved individuals (see Methods for specific sample sizes per analyses). This broad time span covers adolescence, a key period when mental health problems such as depression first emerge (Rapee et al., 2019), and thus also a key period to examine explanatory factors that may help prevent the development of mental health problems. Our main research question was whether the relation between bullying victimization in early adolescence (age 13 years, Wave 2 in TRAILS, T2 in the present study in line with previous TRAILS publications) and depression symptoms in late adolescence (age 19 years, Wave 4/T4) could be explained by victims' interpersonal style in middle adolescence (age 16 years, Wave 3/T3; see Figure 1). More specifically, we expected that victims had a more hostile and less assertive interpersonal style compared to non‐involved peers (as indicated by the positive and negative signs in Figure 1, respectively) which in turn explain victims' increased risk for depression symptoms. Of note, given the contents of the TRAILS database, we were able to account for baseline depression symptoms but not for baseline interpersonal style. This means that we were only partly able to test a temporal causal mediation model. Nonetheless, the TRAILS database was considered to have multiple advantages over previous studies on this topic, as mentioned earlier, thereby adding to the existing literature.

One additional strength of the TRAILS database is that it allowed us to test the bullying victimization–depression link both for self‐reported and peer‐reported bullying victimization nominations. Studies suggest a differential effect between the two nomination types, with self‐reported victimization to be more strongly related to internalizing symptoms such as depression than peer‐reported victimization (Christina et al., 2021). Another strength of the TRAILS database is that it also allowed us to test our mediation model for bully‐victims and bullies. Previous research reported bully‐victims and bullies to also have increased hostility and more depression symptoms relative to non‐involved individuals (e.g., Lereya et al., 2015). For the sake of clarity and structure, we only discuss mediation analyses for the self‐reported victim group in the main manuscript. Nonetheless, we refer the reader to the Supplementary materials for the results and discussion based on peer‐nominations and all respective analyses for the bully‐victim and bully groups.

## METHODS

### Participants and procedures

We used data from the second (T2), third (T3), and fourth (T4) waves of TRAILS. The first wave (T1) took place in 2001–2002, and subsequent waves took place bi‐ or triennially. Of the 135 initially approached schools, 122 agreed to participate. Both parents and children provided informed consent for study participation. The study was approved by the Dutch Central Committee on Research Involving Human Subjects. More detailed descriptions of the recruitment and assessment procedures used in TRAILS and of the representativeness of its sample have been published previously (Huisman et al., 2008; Oldehinkel et al., 2015; Ormel et al., 2012).

A total of 2229 adolescents participated at T1 (76% response rate, *M*_age_ = 11.1 years, 51% female; throughout the waves, gender was assessed with binary (female/male) categories), 2149 at T2 (*M*_age_ = 13.6 years, 51% female), 1816 at T3 (*M*_age_ = 16.3 years, 52% female), and 1881 at T4 (*M*_age_ = 19.1 years, 52% female). A subsample also took part in a peer‐nomination assessment which included peer‐ratings about bullying experiences (see below). For T2, this applied to 1007 participants.

For an overview of descriptive information of participants included in the present study, please see Table 1. Data on self‐reported bullying victimization at T2 was available for 2073 individuals in the whole sample whereas data on peer‐reported bullying victimization at T2 was available for 968 in the subsample.

**TABLE 1:** Descriptive statistics for the whole sample (i.e., based on self‐reported bullying victimization) and subsample (i.e., based on peer‐reported bullying victimization), both for observed and imputed data.

	Whole sample		Subsample	
	Observed data (*N* = 1449)^2^	Imputed data (*N* = 2073)^3^	Observed data (*N* = 717)^4^	Imputed data (*N* = 968)^5^
% female^1^	54.8	51.2	55.2	52.1
By victim status				
Victim	55.6	55.1	53.1	51.4
Bully‐victim	35.7	34.3	26.3	15.0
Bully	36.3	34.6	31.8	28.3
Non‐involved	58.0	53.8	58.0	55.8
T4 age in years (*M* (SD), range)	18.5 (0.60), 17–20	18.6 (0.63), 16.7–20.2	18.5 (0.59), 17–20	18.6 (0.62), 17.0–20.2
By victim status				
Victim	18.5 (0.58), 18–20	18.5 (0.62), 17.2–20.1	18.6 (0.57), 18–20	18.7 (0.63); 17.2–20.0
Bully‐victim	18.5 (0.69), 18–20	18.5 (0.69), 17.6–20.6	18.7 (0.65), 18–20	18.9 (0.67), 17.6–20.0
Bully	18.5 (0.56), 18–20	18.6 (0.61), 17.6–20.0	18.4 (0.62), 17–20	18.6 (0.69), 17.0–20.0
Non‐involved	18.5 (0.60), 17–20	18.6 (0.63), 16.7–20.2	18.5 (0.58), 18–20	18.6 (0.61), 17.4–20.2
T2 Depression symptoms (*M* (SD), range; % sub/clinical)	0.28 (0.24), 0–1.85; 6.49%, 2.35%	0.27 (0.26), 0–1.85; 6.46%, 2.32%	0.28 (0.26), 0–1.77; 3.11%, 2.64%	0.27 (0.31), 0–1.77; 6.00%, 2.58%
By victim status				
Victim	0.52 (0.31), 0–1.77; 12.20%, 5.82%	0.41 (0.31), 0–1.77; 11.31%, 6.20%	0.34 (0.30), 0–1.15; 4.08%, 8.16%	0.32 (0.31), 0–1.15; 5.41%, 8.11%
Bully‐victim	0.48 (0.31), 0–1.08; 17.85%, 10.71%	0.48 (0.31), 0–1.15; 17.81%, 10.96%	0.22 (0.25), 0–0.85; 0.00%, 5.26%	0.23 (0.22), 0–0.85; 2.50%, 2.50%
Bully	0.33 (0.24), 0–1.00; 10.37%, 2.22%	0.31 (0.23), 0–1.08; 9.21%, 1.75%	0.28 (0.29), 0–1.46; 4.55%, 2.27%	0.27 (0.27), 0–1.46; 3.33%, 1.67%
Non‐involved	0.24 (0.24), 0–1.85; 4.40%, 1.31%	0.23 (0.24), 0–1.85; 4.61%, 1.27%	0.28 (0.26), 0–1.77; 6.78%, 2.15%	0.26 (0.26), 0–1.77; 6.42%, 2.14%
T4 Depression symptoms (*M* (SD), range, % sub/clinical)	0.30 (0.30), 0–1.71; 5.24%, 4.21%	0.30 (0.30), 0–1.71; 5.35%, 4.29%	0.30 (0.30), 0–1.71; 5.02%, 4.32%	0.35 (0.32), 0–1.50; 5.68%, 4.24%
By victim status				
Victim	0.37 (0.34), 0–1.57; 9.00, 7.94	0.36 (0.33), 0–1.57; 8.76, 7.66	0.32 (0.29), 0–1.36; 2.04, 2.04	0.35 (0.32), 0–1.50; 5.41, 6.76
Bully‐victim	0.39 (0.32), 0–1.21; 7.14, 7.14	0.35 (0.32), 0–1.21; 5.48, 5.48	0.17 (0.19), 0–0.71; 5.26, 0.00	0.16 (0.22), 0–0.71; 5.00, 0.00
Bully	0.32 (0.32), 0–1.71; 4.44, 7.41	0.32 (0.32), 0–1.71; 6.58, 5.70	0.26 (0.26), 0–1.21; 2.27, 4.55	0.30 (0.27), 0–1.21; 5.00, 8.33
Non‐involved	0.28 (0.28), 0–1.71; 4.58, 2.99	0.28 (0.29), 0–1.71; 4.54, 3.40	0.30 (0.31), 0–1.71; 5.45, 4.46	0.30 (0.31), 0–1.71; 5.79, 3.90
T3 assertiveness (*M* (SD), range)	23.88 (4.51), 8–39	24.02 (4.55), 8.0–39.0	24.16 (4.38), 8–39	23.16 (4.39), 8.0–34.0
By victim status				
Victim	23.05 (4.79), 8–36	22.98 (4.69), 8.0–36.0	22.88 (4.24), 8–32	23.16 (4.39), 8.0–34.0
Bully‐victim	23.20 (4.16), 15–32	23.22 (4.19), 15.0–32.0	23.95 (4.65), 17–32	24.34 (4.54), 17.0–32.6
Bully	24.81 (4.09), 12–38	24.87 (4.20), 11.5–38.0	26.07 (4.04), 17–39	25.59 (4.31), 16.8–39.0
Non‐involved	23.95 (4.51), 10–39	24.12 (4.55), 10.0–39.0	24.13 (4.37), 12–38	24.14 (4.43), 12.0–38.0
T3 Hostility (*M* (SD), range)	20.01 (4.30), 9–38	20.06 (4.37), 7.4–38.0	19.82 (4.29), 9–38	21.39 (5.05), 9.0–38.0
By victim status				
Victim	21.04 (4.60), 10–38	21.17 (4.54), 10.0–38.0	20.90 (5.33), 9–38.0	21.39 (5.05), 9.0–38.0
Bully‐victim	23.02 (4.59), 14–36	23.11 (4.64), 14.0–36.0	20.74 (4.16), 12–29	21.31 (4.27), 12.0–36.4
Bully	21.51 (4.60), 10–33	21.54 (4.45), 10.0–33.0	20.14 (3.75), 11–29	19.78 (4.01), 11.0–29.0
Non‐involved	19.49 (4.05), 9–34	19.48 (4.18), 7.4–34.1	19.68 (4.23), 9–35	19.78 (4.29), 9.0–35.0

*Note*: Means for imputed data are based on pooled means across the 50 imputed datasets. Observed data include individuals that have complete data on all variables relevant for our main analyses. ^1^Gender was assessed with binary (female/male) categories; no other, non‐binary option was available. ^2^ *N*_victims_ = 189, *N*_bully‐victim_ = 56, *N*_bully_ = 135, and *N*_non‐involved_ = 1069. ^3^ *N*_victims_ = 274, *N*_bully‐victim_ = 73, *N*_bully_ = 228, and *N*_non‐involved_ = 1498. ^4^ *N*_victims_ = 49, *N*_bully‐victim_ = 19, *N*_bully_ = 44, and *N*_non‐involved_ = 605. ^5^ *N*_victims_ = 74, *N*_bully‐victim_ = 40, *N*_bully_ = 60, and *N*_non‐involved_ = 794.

### Materials

#### Bullying victimization categories

##### Self‐reported bullying victimization (T2)

We identified four subgroups based on two items of the Youth Self‐Report (YSR; Achenbach & Rescorla, 2001). On a three‐point scale, participants indicated victimization experiences and bullying perpetration in the previous 6 months with items 38 (i.e., “I get bullied a lot”) and 94 (i.e., “I bully others a lot”), respectively; no definition of bullying was provided. Participants who indicated having been victimized 1 = Sometimes or 2 = Often on the first questions and who had 0 = Never according to the second question were categorized as victims. Those who scored 1 or 2 on the perpetration variable and 0 on the victimization variable were categorized as bullies. Scoring 1 or higher on both items were categorized as bully‐victim and scoring 0 on both items were categorized as a non‐involved person.

##### Peer‐reported bullying victimization (T2)

At T2, nominations were assessed in classrooms with at least three TRAILS respondents. TRAILS participants and their classmates were asked to nominate bullies and victims among them; no definition of bullying was provided. There was no limit on the number of nominations. The peer‐nomination assessment took place during school lessons and lasted about 15 min (for more details regarding the peer‐nomination procedure in TRAILS, please see Dijkstra et al., 2009).

Following Jansen et al. (2011), our variable was based on all nominations a TRAILS respondent received from all classmates, divided by the number of respondents in class. This proportion score adjusted for differences in class sizes and T1 victimization (see Jansen et al., 2011 for details), and served as the basis on which we categorized participants.

#### Depression symptoms (T2 and T4)

At T2, depression symptoms in the previous 6 months were assessed with the Affective Problems Scale (APS) of the YSR (Achenbach & Rescorla, 2001). This APS includes 13 items that reflect the symptoms of a major depression episode according to the DSM‐IV (cf. Achenbach & Rescorla, 2003). Participants rated how often they experienced these symptoms on a 3‐point scale (ranging from “0 = Never” to “2 = Often”). At T4, depression symptoms in the previous 6 months were assessed with the APS of the Adult Self‐Report (ASR; Achenbach & Rescorla, 2003); it includes 14 items.

For descriptive purposes, we created a mean APS score for both T2 and T4 with a potential range of 0–2. Internal consistency was acceptable at T2 (α = .78) and good at T4 (α = .83). For our mediation analyses, we decided to dichotomize the depression variable given its highly skewed distribution (0 vs. >0), using the official gender‐stratified cut‐offs for both the YSR‐APS and ASR‐APS (see Achenbach & Rescorla, 2001, 2003). A value of 0 represented no depression and 1 represented sub‐ or clinical levels of depression symptoms.

#### Assertiveness and hostility (T3)

Adolescents' interpersonal style was assessed with the NEO‐PI‐R (Costa & McCrae, 1985, 1992). Only a sub‐selection of facets was administered in TRAILS (see Oldehinkel et al., 2015) of which we used assertive‐dominance (α = .75; part of the Extraversion domain) and angry‐hostility (α = .71; part of the Neuroticism domain), labeled assertiveness and hostility in the Introduction. Both scales consisted of eight items which were scored on a 5‐point scale ranging from “1 = Totally disagree” to “5 = Totally agree.” An example item for assertiveness was “I am dominant, forceful and assertive,” and for hostility “I am known as hot‐blooded and quick‐tempered.” Scores as used in our analyses represent total item scores with a potential range of 8–40. More assertiveness is associated with higher dominant behavior and more hostility is associated with more quarrelsome behavior (e.g., Du et al., 2020).

### Planned analyses

Analyses were primarily performed on 2073 participants with self‐reported bullying victimization data (i.e., *N*_victims_ = 274, *N*_bully‐victim_ = 73, *N*_bully_ = 228, and *N*_non‐involved_ = 1498) and secondarily on a subsample of 968 participants with peer‐reported bullying victimization data (*N*_victims_ = 74, *N*_bully‐victim_ = 40, *N*_bully_ = 60, and *N*_non‐involved_ = 794). Below, we describe results based on the self‐reported data, focusing on the comparison between the victim and the non‐involved groups, specifically the mediation analyses. We describe results regarding the peer‐reported data and for results regarding the bully‐victim and bully groups in the Supplementary materials.

As we used data from three time points, some T2 participants had missing data on our key T3 and T4 variables (see Analyses with self‐reported bullying victimization data for details). In order to run our analyses on the largest possible *N*, we performed multiple imputation of the missing data. In this manuscript, we describe results for analyses based on the imputed samples (i.e., 2073 and 968, respectively). Please see Appendix S9 for a description of analyses with the complete observed data (*N*_self‐report_ = 1449 and *N*_peer‐report_ = 717).

Not all of the T2 participants also partook in the following waves of the study. This is why we had missing data for variables used in the primary mediation analyses, namely assertiveness and hostility (T3) and depression symptoms (T4). On the variable level, 21%–22% of the data were missing for assertiveness, hostility, and for T4 depression symptoms. On the participant level, about 8% of participants missed information for one variable, 9% for two variables, and 12% for all three variables; 71% of participants had no missing data. We investigated whether participants with complete versus missing data differed regarding gender, T2 self‐reported or peer‐reported bullying victimization, T2 age, or T2 depression symptoms using chi‐squared‐tests, t‐tests, and Wilcoxon‐Mann–Whitney tests. These baseline analyses were performed in SPSS version 27. The required level of significance (alpha) for all analyses presented was set at .05. Participants with missing data were more likely than participants with complete data to be male, a self‐reported bully, a peer‐reported victim or bully‐victim, slightly older, and have less depression symptoms (see Appendix S1 for test statistics and a detailed description of results).

Data imputation was performed using the mice package in R (van Buuren & Groothuis‐Oudshoorn, 2010). We performed a multiple imputation to complete the amount of missing data for T3 assertiveness and hostility (mediators) and for T4 depression symptoms (outcome variable), but did not impute values for the missing observations in peer reported bullying victimization (T2). We created a custom‐made imputation model (cf. van Buuren, 2018) to accommodate both normally and non‐normally distributed variables (see Table S1 in Appendix S2 for an overview of the imputation procedure). A total of 50 imputed datasets were created. An overview of descriptive statistics for relevant study variables based on the original and the imputed data can be found in Table 1, and respective Pearson correlation coefficients in Table S2 in Appendix S3.

With our dichotomized outcome variable T4 depression, we performed logistic regression analyses. Mediation analyses were performed according to the potential outcomes framework (Imai, Keele, & Tingley, 2010; Imai, Keele, & Yamamoto, 2010). Compared to the well‐known structural equation modeling approach (i.e., Baron–Kenny), the potential outcomes framework offers a different approach to estimating the direct and indirect effect, and is particularly useful given that our sample was divided into multiple bullying victimization categories/groups. In addition, it can be used to test multiple mediator effects simultaneously. For a detailed explanation of the advantages of the potential outcomes framework and how it compares to the Baron‐Kenny method, we refer the reader to Appendix S4.

We performed the path mediation analyses separately for each of the 50 imputed datasets. Per imputed dataset, indirect effects were computed based on 1000 bootstrapped samples. To obtain the overall mediation (indirect) effect over the 50 imputed datasets, we averaged the estimates, and obtained 95% adjusted bootstrap percentile confidence intervals and *p*‐values. Moreover, the proportion of the direct effect mediated (i.e., taken over by the mediators, through their indirect effect) was estimated. This proportion can be used for assessing the mediation effect size. The obtained estimates are marginal, that is, averaged over the respondents, while taking into account their covariate values, and thus provide the overall mediation effect. Figure 1 graphically represents the model.

We tested whether T3 assertiveness and hostility mediated the association between T2 bullying victimization groups and T4 depression symptom levels in one model, including the two mediators simultaneously. Gender, (grand‐mean centered) T4 age, and (dichotomized) T2 depression symptoms were included in the model as covariates. Our T2 predictor was dummy‐coded, comparing the victim group (D1), the bully‐victim group (D2), and the bully group (D3) to the non‐involved group, respectively. The inclusion of T2 depression symptoms as a covariate served as safeguard against overestimation of the mediation effects. Both mediators were grand‐mean centered.

Here, we report the results of the mediation analyses by focusing on the comparison between the victim and non‐involved group (for a textual description of the results of the other mediation analyses see Appendix S7; for test statistics based on imputed data see Table 2 and based on observed data see Table S5 in Appendix S10). The report consists of two steps. First, the total and direct effects are reported, starting from the descriptive differences in the probability of having depression symptoms, then reporting this difference after taking into account the effect of the covariates, followed by the effects of either mediator alone and of both mediators together. Second, we report the indirect effects, that is, the difference in the probability of having depression symptoms between victims and non‐involved individuals, due to each of the two mediators. This difference is expressed as a percentage of the total difference in probabilities after taking into account the effects of the covariates and the other mediator. Thus, a “net” mediation effect was obtained, both absolute (as the difference in probabilities) and relative (as the percentage of the total effect). We also tested whether there were differential effects of the two mediators on depression symptoms for the bullying victimization groups. In other words, we examined the bullying victimization groups by mediator interaction on depression symptoms. In case of a significant group by mediator interaction, we described the respective differential mediation effects in the text.

**TABLE 2:** Estimated mediation effects of hostility and assertiveness on probability for depression symptoms dummy‐coded, comparing victims with non‐involved (NI) as reference group; based on imputed data.

Victims	Self‐report				Peer‐report			
	Hostility		Assertiveness		Hostility		Assertiveness	
	Estimate	*p*	Estimate	*p*	Estimate	*p*	Estimate	*p*
Indirect effect (a*b)	**0.013**	**.0001**	Victim: −0.004 **NI: 0.006**	Victim: .331 **NI: .010**	0.006	.470	0.004	.224
Averaged direct effect	0.030	.164	0.030	.164	−0.027	.505	−0.027	.507
Total effect	0.042	.050	0.036	.088	−0.021	.587	−0.023	.560
Proportion mediated	0.312	.051	0.020	.773	0.030	.803	−0.112	.602

*Note*: Significant mediation effects at an alpha of .05 are highlighted in bold.

## RESULTS

### Bullying victimization prevalence rates

Using self‐reported bullying information, about 13% of participants considered themselves a victim, 4% a bully‐victim, 11% a bully, and 72% a non‐involved person. As for the overlap between self‐ and peer‐reported nominations, there was a considerable discrepancy (see Appendix S5 for details).

### Depression symptoms of victims and non‐involved peers

Overall, when simply examining the distribution of having depression symptoms by bullying victimization nomination using a crosstab, victims had a .164 probability for depression symptoms at T4, which is .085 higher compared to non‐involved individuals (.079).

### Associations between self‐reported bullying victimization, hostility, assertiveness, and depression symptoms

When we continued to examine whether hostility and assertiveness were justified to be tested as mediators of the bullying‐depression relation, we found an overall statistically significant group difference regarding hostility (*F*(3, 1024.8) = 23.04, *p* < .001) and assertiveness (*F*(3, 1004.0) = 6.94, *p* < .001), when accounting for the effect of the covariates. Post‐hoc regression analyses (see Table 3) showed victims (*M* = 21.17, SD = 4.54) having significantly more hostility than the non‐involved group (*M* = 19.48, SD = 4.18). Concerning assertiveness, there was one significant group difference. Victims (*M* = 22.98, SD = 4.69) had significantly lower assertiveness than non‐involved individuals (*M* = 24.12, SD = 4.55).

**TABLE 3:** Summary of multiple linear regression analyses for variables predicting hostility and assertiveness based on self‐reported bullying victimization and pooled across the 50 imputed datasets (*n* = 2073).

Variable	Hostility			Assertiveness		
	*B*	SE	*p*	*B*	SE	*p*
Intercept	−0.44	0.16	.005	−0.13	0.16	.418
Victim vs. NI^a^	**1.04**	**0.31**	**<.001**	**−0.92**	**0.34**	**.006**
Bully‐victim vs. NI^a^	**2.73**	**0.58**	**<.001**	−0.80	0.60	.182
Bully vs. NI^a^	**2.01**	**0.41**	**<.001**	0.67	0.38	.074
Gender (1 = male)	−0.55	0.21	.010	0.72	0.23	.001
T4Age	0.11	0.18	.535	−0.05	0.20	.968
T2Depr^b^	3.00	0.38	<.0001	−1.24	0.40	.002
Adj. R^2^	.086		<.05	.025		<.05

Abbreviation: NI, non‐involved comparison group.

^a^ Dummy coded bullying victimization groups.

^b^ Dichotomized depression symptoms at T2 with 0 = non‐clinical and 1 = sub‐or clinical symptom levels. The most relevant (based on hypothesis testing) significant effects, based on an *⍺* of .05, are highlighted in bold.

We then examined how the differential probability for depression symptoms between the bullying victimization groups and the non‐involved group changed once accounting for only the covariates, then also for one of the mediators, and then also for the second mediator. For descriptive purposes, we used a linear (not logistic) regression model, to provide rough estimates on the probability scale (which were all significant). Focusing on the victims, when taking the effect of the covariates into account, victims had an increased probability of .051 of having depression symptoms compared with non‐involved individuals. When also accounting for the effect of hostility, the increased probability of victims' depression symptoms was .038. Comparably, when accounting for the effect of assertiveness and the covariates, victims had a .047 higher probability for depression symptoms over non‐involved individuals. Finally, taking the effects of both mediators and the covariates into account, being a victim of bullying compared to a non‐involved, meant a .034 higher chance for T4 depression symptoms. These findings gave a first indication that, while being a victim certainly increases the risk for depression symptoms, a sizable part of the increase can be accounted for by the mediators, especially hostility (because adding it as a covariate reduced the probability by .013 while adding assertiveness as a covariate only reduced the probability by .004). We further examined this relation with more precision by means of mediation analyses.

### Mediation of hostility on the victim–depression relation

For an overview of the tested mediation for the victim group, including respective statistics, please see Figure 1 and Table 2. For a textual description of the tested mediation for the peer‐reported victim group see Appendix S5, and for the bully‐victim and bully groups (based on both nomination styles) please see Appendices S7 and S8.

The indirect effect of the victim dummy was significant (*p* = .0001) and positive (a*b = 0.013). Relative to the total effect (0.042; *p* = .050), when accounting for the covariates and assertiveness, the estimated proportion mediated of the effect of being a victim compared to a non‐involved individual on depression symptoms due to hostility was estimated as 31.2% (*p* = .051). The estimates and *p*‐values are based on the pooled average proportion obtained by a bootstrap sample for each of the 50 imputed datasets. The precision of the estimated proportion warrants the interpretation that almost one‐third of the total effect was due to hostility, which we consider a moderate effect.

### Mediation of assertiveness on the victim–depression relation

We found a significant victim dummy by assertiveness interaction effect, implying that the mediation effect differed for the victim and non‐involved groups. Therefore, rather than reporting the averaged mediation estimate, we report separate mediation effects for the victim group and the non‐involved group. For the victim group, the mediation effect for assertiveness was not significant (a*b_Victims_ = −0.004; *p*_Victims_ = .331). Thus, assertiveness did not explain victims' risk for depression symptoms. For the non‐involved group, the mediation effect was significant (a*b_non‐involved_ = 0.006; *p*_non‐involved_ = .010). However, the proportion of this mediation was .020 meaning that only 2% of non‐involved individuals' higher likelihood for depression was explained by their higher assertiveness.

## DISCUSSION

In the present study, we utilized data from the large longitudinal population‐based study TRAILS to examine the role of assertiveness and hostility in explaining the increased risk for depression symptoms in victims of bullying compared to peers without bullying experiences. We tested this association twice, using either self‐reported (see below) or peer‐reported (see Appendix S5) bullying victimization information. In secondary analyses, we also examined our mediation model for bully‐victims and bullies (see Appendix S7 for a detailed discussion).

### Mediation of the self‐reported victimization–depression link by hostility but not assertiveness

In line with previous research (e.g., Camerini et al., 2020; Reijntjes et al., 2010; Salmivalli & Helteenvuori, 2007; Salmivalli & Nieminen, 2002), we found that self‐reported victims had an increased risk for depression symptoms and had more hostile traits than non‐involved individuals. In addition, we found that hostility explained nearly a third of victims' increased depression risk compared to non‐involved peers. In other words, our results suggest that individuals who, at the age of 13 years, describe themselves as victims of bullying have a more hostile interpersonal style by mid‐adolescence (age 16 years) which in turn contribute to more depression symptoms by the age of 19 years.

The concept of interpersonal complementarity can help to better understand how exactly hostility may over time contribute to (long‐term) interpersonal struggles and to an increased risk for depression. Repeated encounters with aggressive, dominant bullies can increase hostile behavior by victims, possibly as a way to defend themselves. In turn, victims' hostility might invite bullies to re‐victimize them (cf. Sadler et al., 2011). Repeated negative encounters with bullies can also lead to victims more generally feeling unsafe in social situations, losing trust in others, and becoming more suspicious (i.e., increased hostile attributions and perceptions; Jantzer et al., 2006; Kellij et al., 2022; Nepon et al., 2021; Zimmer‐Gembeck, 2016). As a consequence, victims might also act more hostile to individuals other than bullies who in turn might also respond with hostility (cf. Moskowitz, 2010). This means that over time, victims may experience increasingly more interpersonal conflicts which impacts the quality of their interpersonal relationships and the social support they receive from friends, family, and partners. Interpersonal problems and lack of social support are thought to represent risk factors for depression (e.g., Vrshek‐Schallhorn et al., 2015).

As we were not able to account for baseline interpersonal style, our results cannot be used to draw directional conclusions regarding the relation between victimization and hostility. Indeed, we still assume there to be a bidirectional relation between victimization and interpersonal style, as this has also been suggested in previous studies (Kljakovic & Hunt, 2016; Schoeler et al., 2018). Nonetheless, thanks to the longitudinal nature of our data and being able to statistically control for baseline depression symptoms, we can with fair confidence conclude that the risk for depression increases as a consequence of victims' hostility.

In line with previous research (e.g., Fox & Boulton, 2006), victims were also found to be less assertive than non‐involved peers. However, assertiveness was not a significant mediator of the victimization‐depression relation. Assertiveness exclusively explained the (generally low) risk for depression symptoms of non‐involved individuals, and only to a very small extent.

Thus, while hostility helped explain how self‐reported victims of bullying may develop depression symptoms, we did not find comparable evidence for a lack of assertiveness as a mediator of the victimization‐depression link. It has been argued that people are more “socially allergic” to hostility compared to non‐assertiveness and more readily interpret a hostile style as aversive (O'Connor, 2011). Hostility in victims might therefore more often lead to interpersonal conflicts and rejection than a lack of assertiveness. This might then explain why high hostility but not low assertiveness was a mediator of victims' depression risk.

Of note, for peer‐reported victimization, neither hostility nor assertiveness significantly explained victims' risk for depression symptoms. This implies that the outcome of our mediation model differed depending on whether the person themselves or peers were the information source for the bullying victimization experiences. Overall, the difference in findings was not unexpected. Existing research has previously shown that self‐reported victimization tends to exhibit stronger associations with depression compared to victimization reported by peers (e.g., Pouwels et al., 2016). We discuss the discrepancy in findings in detail in Appendix S5.

### Study limitations

Although we took advantage of the population‐based longitudinal TRAILS database, used two different bullying information sources (self‐ and peer‐reports), and applied advanced statistical methods to analyze our data, our study is not without limitations. As already discussed in and similar to previous TRAILS publications (e.g., Kaufman et al., 2020; Kretschmer et al., 2018), self‐reported bullying victimization was assessed using two questions only. Although still considered useful in characterizing bullying experiences (Furlong et al., 2010), a multiple‐item assessment using a standardized bullying questionnaire which also provides a precise definition of bullying is considered more valid (Thomas et al., 2015). Similarly, even though we adhered to previous TRAILS work, our peer‐reported victimization categorization had its drawbacks (i.e., more conservative categorization criteria for victims as it was also adjusted for T1 victimization identical to Jansen et al. (2011)).

Participants' hostility and assertiveness were assessed using the assertive‐dominance and the angry‐hostility facets of the NEO‐PI‐R which in TRAILS were not explicitly intended to be used as measures of interpersonal style. Other, more suitable questionnaires exist as well (cf. Locke, 2011). An example is the Revised Interpersonal Goals Inventory for Children (IGI‐CR; Trucco et al., 2013) which has also been used in a sample with bullying experiences (cf. Dollar, 2016). Nevertheless, the used NEO‐PI‐R facets have shown to be a reliable and valid representation of interpersonal style (Du et al., 2020).

### Future directions

Internalizing problems such as depression have been established as both antecedents and consequences of bullying victimization (Reijntjes et al., 2010). This hints toward a vicious cycle perpetuating both victimization and internalizing problems. Nonetheless, multiple studies have found that being a victim of bullying contributes uniquely to the later development of mental‐health problems (Arseneault, 2018; Ttofi et al., 2011). We based our mediation model on this directional association.

However, following previous research (Arseneault et al., 2010), depression symptoms can also predict later victimization, suggesting a reverse mediation model. As it was not possible to test this model in the TRAILS data, future studies should also investigate whether depression symptoms can explain the risk for experiencing bullying victimization through interpersonal differences. Based on the notion that both mediation models hold true, a difference in the strength of effects could guide interventions, and suggest when and for whom addressing interpersonal style differences is most helpful.

In addition, future research could also test the applicability of our model for diagnosed depression. Our findings are based on depression symptoms in the sub‐ and clinical range but are not based on clinical diagnoses. Research in the field of depression has suggested interpersonal skills to be predictive of not only symptoms but also diagnosable depression (Gadassi & Rafaeli, 2015). Therefore, victims' (and actually also bully‐victims' and bullies') hostile traits may also explain their depression diagnosis, and thus, our model could also be applicable to understand how they develop more severe types of this internalizing disorder. The strength of our proposed mediation would likely be stronger as depression symptoms become more intense or clinical.

### Implications

We found that interpersonal traits, specifically high hostility, can help explain victims' increased risk for depression symptoms. This finding was based on self‐reported bullying victimization experiences. This suggests that asking an adolescent directly about whether or not they feel bullied serves as a better predictor of which interpersonal traits they might develop and whether they subsequently develop depression symptoms, than relying on other‐reports. Therefore, teachers need to make sure to take the child's own perception seriously, even if they themselves or peers do not consider the child a victim of bullying (cf. Oldenburg et al., 2016).

Following our longitudinal findings, support for a victimized adolescent could help prevent adjustment problems and mental health problems later in life. Adolescence is a critical period for identifying and addressing emotional and behavioral problems, which may help develop protective factors and prevent risk factors that contribute to the onset of serious mental health problems. As a critical test of our mediation findings with respect to their implied causality, interventions could focus on offering emotion regulation techniques and social skills training in order for victims to learn how to express frustration and anger more adaptively, and therefore avoid the development of hostile traits. This way, interpersonal conflicts and adjustment problems could be reduced and therefore also the development of depression in the long‐term (at least to a certain degree).

## CONCLUSIONS

We used a large longitudinal population‐based database to test if interpersonal characteristics can help explain the increased risk for depression symptoms in victims of bullying as compared to peers without involvement in bullying. Overall, findings indicated that about a third of the depression risk of (self‐reported) victims can be explained by their relatively high hostility. Similar findings were observed for bully‐victims and bullies (see Appendix S7), suggesting that, overall, the high hostility that may characterize interactions between bullies and victims may play a central role in increasing their risk for depression and other internalizing problems.

## CONFLICT OF INTEREST STATEMENT

The authors declare none.

## INFORMED CONSENT

Both parents and children provided informed consent for study participation.

## Supporting information


Appendices S1–S10.

## Data Availability

Data available on request due to privacy/ethical restrictions.
