# Supplementary material for: How do victims of bullying develop depression? Testing interpersonal style to explain the victimization‐depression link
Source: J Res Adolesc. 2024 Jul 23;34(4):1391–402. doi: 10.1111/jora.13005 (PMC11606264; doi:10.1111/jora.13005)
Supplement: Supplementary file 1 — Appendices S1–S10. [file JORA-34-1391-s001.docx]

**Appendix S1**

**Analyses of Missingness**

We tested whether participants with complete data (*N*= 1449) and missing data (*N* =624) differed in various T2 variables. The relation between having missing data and gender was significant (χ^2^(1) 25.17, *p*<.0001); 57% of individuals with missing data were male whereas only 45% of individuals with complete data were male. The distribution of self-reported victim status differed between the missing and complete group (χ^2^(3,)= 15.62 *p*=.001). There were 74% non-involved individuals, 13% victims, 4% bully-victims, and 9% bullies in the complete group. In comparison, there were fewer non-involved (69%), about equal victims (14%) and bully-victims (3%), and more bullies (15%) in the missing group. The two groups also differed in the distribution of peer-reported victim status (χ^2^(3)= 18.97 *p*=.0003). There were 84% non-involved individuals, 7% victims, 3% bully-victims, and 6% bullies in the complete group. In comparison, there were fewer non-involved (75%), and more victims (10%) bully-victims (8%), and bullies (15%) in the missing group. Participants with missing data were slightly older (*M*= 13.15, *SD*= 0.61) than those without missing data (*M*= 13.02, *SD*= 0.60; *t*(2071)= -4.55, *p*< .0001). Individuals with missing data (*Median*= 0.15) had less depression symptoms than individuals who did not miss any data (*Median*= 0.23; *Z*= -3.27, *p*=.001). To summarize, participants with missing data were more likely to be male, a self-reported bully, a peer-reported victim or bully-victim, slightly older, and have less depression symptoms.

**Appendix S2**

| Supplementary Table S1 |  | | | | | | |
| --- | --- | --- | --- | --- | --- | --- | --- |
| *Variables and their roles involved in the multiple imputation procedure* | | | | | | | |
|  | **Imputed variables**  **(method of imputation)** | | | | | | |
|  | **Main variables** | | | | **Helping variables for imputation** | | |
|  |  |  |  |  |  |  |  |
|  | Assert (LINReg) | Host  (LINReg) | T4Age (LINReg) | T4Depr  (PMM) | PR- bullying victimizationgroups (Polyreg) | T3Age (LINReg) | T3Depr (PMM) |
| **Variables used for imputation** |  |  |  |  |  |  |  |
| SR-bullying victimization groups^A^ | 1 | 1 | 1 | 1 | 1 | 1 | 1 |
| PR- bullying victimization groups^A^ | 1 | 1 | 1 | 1 | 0 | 1 | 1 |
| Assert | 0 | 1 | 1 | 1 | 1 | 1 | 1 |
| Host | 1 | 0 | 1 | 1 | 1 | 1 | 1 |
| Gender | 1 | 1 | 1 | 1 | 1 | 1 | 1 |
| T4Age | 1 | 1 | 0 | 1 | 1 | 1 | 1 |
| T4Depr | 1 | 1 | 1 | 0 | 1 | 1 | 1 |
| T2Age | 1 | 1 | 1 | 1 | 1 | 1 | 1 |
| T3Age | 1 | 1 | 1 | 1 | 1 | 0 | 1 |
| T2Depr | 1 | 1 | 1 | 1 | 1 | 0 | 0 |
| T3Depr | 1 | 1 | 1 | 1 | 1 | 1 | 0 |
| SR-bullying victimization groups^A^ * Assert | 0 | 1 | 1 | 1 | 1 | 1 | 1 |
| SR- bullying victimization groups^A^ * Host | 1 | 0 | 1 | 1 | 1 | 1 | 1 |
| PR- bullying victimization groups^A^ * Assert | 0 | 1 | 1 | 1 | 0 | 1 | 1 |
| PR- bullying victimization groups^A^ * Host | 1 | 0 | 1 | 1 | 0 | 1 | 1 |
| Gender * Assert | 0 | 1 | 1 | 1 | 1 | 1 | 1 |
| Gender * Host | 1 | 0 | 1 | 1 | 1 | 1 | 1 |
| T2Depr * Assert | 0 | 1 | 1 | 1 | 1 | 1 | 1 |
| T2Depr * Host | 1 | 0 | 1 | 1 | 1 | 1 | 1 |
| Gender * T3Depr | 1 | 1 | 1 | 1 | 1 | 1 | 0 |
| T2Age * Assert | 0 | 1 | 1 | 1 | 1 | 1 | 1 |
| T2Age * Host | 1 | 0 | 1 | 1 | 1 | 1 | 1 |
| T3Age * Assert | 0 | 1 | 1 | 1 | 1 | 0 | 1 |
| T3Age * Host | 1 | 0 | 1 | 1 | 1 | 0 | 1 |
| Gender * T2Depr | 1 | 1 | 1 | 1 | 1 | 1 | 1 |
| *Note*: ^A^ dummy coded with the non-involved group as comparison group. 1 = used in imputation. PMM= predictive mean matching; used to impute variables with a skewed distribution. LINReg= linear regression model; used to impute symmetrically distributed variables. POLYReg= polynomial regression model; used to impute categorical variables. SR = self-reported. PR = peer-reported. Depr = Depression symptoms. Assert = assertiveness. Host = hostility. The following variables were grand-mean centered: Assertiveness, hostility, age (T2-T4), T2 and T3 depression. | | | | | | | |

| Supplementary Table S2. | | | | | | |
| --- | --- | --- | --- | --- | --- | --- |
| *Pearson correlation coefficients for self-reported/peer-reported study variables based on observed data (lower triangle) and imputed data (upper triangle).* | | | | | | |
| Variables | 1. | 2. | 3. | 4. | 5. | 6. |
| 1. T1 female |  | **-.058/-**.061 | **.187/.159**  .030/.006 | **.212/.199**  **.091/.088** | **-.087**/-.053 | **.047**/.060 |
| 1. T4 age | -.026/-.033 |  | -.040/-.034  .001/.030 | .030/.051  .032/.055 | -.001/.025 | .006/-.004 |
| 1. T2 depression symptoms | **.166/.153**  **.019/.016** | -.005/.023  .029/.066 |  | **.420/.404**  **.210/.194** | **-.122/-.131**  **-.091/-.082** | **.325/.347**  **.225/.234** |
| 1. T4 depression symptoms | **.202/.191**  **.090/.096** | .040/**.077**  .041/.068 | **.442/.405**  **.207/.168** |  | **-.126/-.145**  **-.088/-.102** | **.328/.324**  **.225/.243** |
| 1. T3 Assertiveness | **-.070**/-.042 | -.013/.015 | **-.117/-.135**  **-.088/-.073** | **-.125**/**-.147**  **-.092/-.106** |  | -.005/-.054 |
| 1. T3 Hostility | **.058**/**.085** | .009/-.001 | **.349/.369**  **.235/.238** | **.346/.333**  **.235/.252** | -.033/**-.078** |  |
| *Note*. Values significant at an alpha of .05 are represented in bold. Values for imputed data (upper triangle) are based on pooled estimates across the 50 imputed data sets. The upper row values for T2 and T4 depression symptoms are based on the continuous scores while the lower row values are based on the respective dichotomous (non-clinical vs. sub-or clinical) depression scores. | | | | | | |

**Appendix S3**

**Appendix S4**

**Advantages of the Potential Outcomes Framework and How it Compares to the Baron-Kenny Method for Performing Mediation Analyses**

The potential outcomes framework defines the effect of a ‘treatment’ on an outcome variable as the (expected) difference between two ‘potential’ outcomes (treated vs. control), where one of the two potential outcomes is observed and the other is not. Examining the role or effects of mediators, measured between treatment and outcome, fits well in this framework. The indirect effect is defined as the (expected) difference in potential outcomes in the same treatment group (thus eliminating the direct effect), one with the values of the mediator for the treated group, and the other with the values for the mediator for the control group (cf. MacKinnon et al., 2020; Valeri & VanderWeele, 2013).

The analyses were performed using the mediation package in R (Tingley et al., 2014), comparing each of the three ‘treated’ groups, victims, bullies and bully-victims, to the ‘control’ group of non-involved participants. Compared to the more often used Hayes PROCESS macro in SPSS (Hayes, 2012), which is based on the Baron-Kenny approach, the mediation package in R has at least three advantages for the type of analyses and models used in the present study. Firstly, it provides good parameter estimates for models that include categorical predictors and dichotomous outcome variables (Tingley et al., 2014). Secondly, it presents the estimates of the predictor (that is, the differences between the non-involved (reference/control) group and one of the three other bullying victimization groups) on a probability outcome scale as opposed to log-odds, which facilitates the interpretation of results. Thirdly, the mediation package allows for testing for differential mediator effects, i.e. not assuming that the indirect effect is equal for both (i.e., ‘treated’ and ‘control’) groups. This implies that a group by mediator interaction is estimated which, when significant, is included in the model. This would then lead to improved model fit and more precise mediation effects. Finally, it is possible to test multiple mediator effects simultaneously, provided that the mediators are independent from each other (Imai et al., 2010).

**Appendix S5**

**Analyses with Peer-reported Bullying Victimization Data.**

We repeated the mediation analyses as described in the main manuscript for the imputed data (*N*= 986) and the original observed data (*N*= 717) for a sub-sample for which T2 peer-reported bullying victimization data were also available.

**Bullying Victimization Prevalence Rates**

Peers nominated about 8% of their classmates as victims, 4% as bully-victims, 6% bullies, and 82% to be non-involved. In comparison, using self-reported bullying information, about 13% of participants considered themselves a victim, 4% a bully-victim, 11% a bully, and 72% a non-involved person.

There was a considerable discrepancy between self- and peer-reported nominations. Only 19% of self-reported victims were also reported as victims by their peers; most of the peers reported them as non-involved (73%), and a minority as bully-victims (6%) or bullies (2%). Similarly, only 10% of self-reported bully-victims were also reported as bully-victims by their peers; most of them (66%) were reported as a non-involved person by their peers, 21% as victims, and 3% as bullies.

Conversely, of those individuals who were peer-rated as victims, only 32% also rated themselves as victims, whereas about half (53%) indicated to be not involved (8% indicated to be a bully-victim, and 7% to be a bully). The overlap between ratings was even lower for peer-reported bully-victims. Only 8% of them also indicated to be a bully-victim. Most of them indicated to be a non-involved person (55%), 20% to be a bully, and 17% to be a victim.

**Depression Symptoms per Bullying Victimization Group**

Peer-reported victims had a probability of .122, bully-victims of .133, bullies of .050, and non-involved individuals of .097 for depression symptoms at T4.

**Associations Between Peer-reported Bullying Victimization, Hostility, Assertiveness, and Depression Symptoms**

There was no clear group difference regarding hostility (*F*(3,586.1)= 2.51, *p* = .057) and assertiveness (*F*(3,558.2)=2.04 , *p* = .106) when accounting for the effect of the covariates. Post-hoc regression analyses (see Supplementary Table S3 in Appendix S6) indicated that neither victims, nor bully-victims or bullies were significantly different in their hostility from the non-involved group. Regarding assertiveness, only the bully group significantly differed from the non-involved group, having a higher mean.

When we examined how the differential probability for depression symptoms between the three bullying victimization groups and the non-involved group changed once accounting for the covariates, one of the mediators, and both mediators. Focusing on the victims, when taking the effect of the covariates into account, victims had a decreased probability of -.013 of having depression symptoms compared to non-involved individuals. When also accounting for the effect of hostility, the decreased probability of victims’ depression symptoms was -.026. When accounting for the effect of assertiveness and the covariates, victims had a -.019 lower probability for depression symptoms than non-involved individuals. Finally, taking the effects of both mediators and the covariates into account, being a victim of bullying rather than a non-involved, meant a -.030 lower chance for T4 depression symptoms. These findings indicate that peer-reported victim nomination explains the decreased risk for depression symptoms to a certain degree, with hostility accounting for more than assertiveness (because adding hostility as a covariate reduced the probability further by 0.013 while when adding assertiveness as a covariate it only reduced the probability further by 0.005).

**Mediation of Hostility and Assertiveness on the Peer-reported Victim-Depression Relation**

Table 3 includes test statistics for the respective mediation analyses. The indirect effect for neither hostility (*p*= .470) nor assertiveness (*p*= .224) was significant. Therefore, none of the mediators significantly contributed to explaining the decreased risk for peer-reported victims to develop depression symptoms.

**Discussion of Results**

**Comparison Between Mediation Models Using Peer-reported vs Self-reported Victimization**

When indexing victimization on the basis of peer-reports, neither hostility nor assertiveness significantly explained victims’ risk for depression symptoms. This means that the outcome of our mediation model differed depending on the information source for the bullying victimization experience. In general, this discrepancy between findings in self-reported and peer-reported victims was not unexpected. Previous literature has reported stronger associations with depression for self-reported than peer-reported victimization (e.g., Pouwels et al., 2016).

The differences in findings may be explained by the limited overlap between the bullying victimization nomination types. About three-quarter of self-reported victims were peer-reported as non-involved individuals, whereas half of the peer-reported victims labelled themselves as not having had any bullying experiences. As already discussed in previous literature (e.g., Hwang et al., 2017; Pouwels et al., 2016), this discrepancy in self-reported versus peer-nominated bullying victimization can have various reasons. Firstly, peers might not always see the bullying taking place. The older children become, the subtler and more covert the bullying becomes (Casper & Card, 2017), which might therefore be less likely to be noticed by peers. Secondly, individuals who consider themselves a victim but are not rated as such by their peers might misinterpret social situations more often as hostile, and as involving bullying than other peers (cf. Hwang et al., 2017; Pouwels et al., 2016). This in turn would lead to a self- but not a peer-reported victim nomination. Biased processing of social information has also been suggested as an explanation for why self-reported victims generally report more depression symptoms than peer-reported victims (e.g., Perren et al., 2013; Zimmer‐Gembeck, 2016). Thirdly, individuals who are peer-nominated as victims but do not rate themselves as victims might either not feel comfortable to report their victimization experiences or might not interpret the behavior of others as bullying (Hwang et al., 2017). In the latter case, those individuals might be less impacted by their bullying experiences and therefore also experience less negative consequences. This might explain why our peer-reported victim group had a lower risk for depression than the peer-reported non-involved group (which included many self-reported victims).

The difference in findings might also be due to a difference in perceived stress. Depression is considered an internalizing disorder with, for example, cognitive biases negatively influencing social information processing, which increases the risk for depression symptoms (see e.g., Everaert et al., 2017). The subjective (i.e., self-reported) experience of being victimized might, therefore, be a stronger predictor of depression symptoms compared to peer-nominations of victimization (which might not overlap with personal experience). This is also suggested by the ecological-transactional model (Zimmer‐Gembeck, 2016), which suggests that victims’ social perceptions, which includes the awareness and sensitivity of rejection by peers, disregarding or even despite others reporting no such victimization, to be a critical factor explaining mental health problems. Victims that are both self- and peer-identified have more internalizing symptoms compared to self-reported-only (and also compared to peer-reported-only) victims (Scholte et al., 2013). As has also been pointed out previously (e.g., Bouman et al., 2012; Pouwels et al., 2016), the differential findings for self- and peer-reported victimization need to be considered when examining the mental health problems of individuals with bullying experiences.

| Supplementary Table S3. | | | | | | |
| --- | --- | --- | --- | --- | --- | --- |
| *Summary of multiple linear regression analyses for variables predicting hostility and assertiveness based on peer-reported bullying victimization and pooled across the 50 imputed data sets (n=968).* | | | | | | |
|  | Hostility | | | Assertiveness | | |
| Variable | B | SE | p | B | SE | p |
| Intercept | -0.34 | 0.22 | .119 | 0.11 | 0.22 | .612 |
| Victim vs. NI^A^ | -0.87 | 0.59 | .141 | -0.90 | 0.62 | .147 |
| Bully-victim vs. NI ^A^ | 1.66 | 0.93 | .076 | -0.07 | 0.91 | .931 |
| Bully vs. NI ^A^ | 0.72 | 0.65 | .264 | **1.60** | **0.71** | **.024** |
| Gender (1= male) | -0.65 | 0.30 | .029 | 0.36 | 0.31 | .250 |
| T4Age | -0.11 | 0.25 | .673 | 0.21 | 0.26 | .431 |
| T2Depr ^B^ | 3.65 | 0.54 | <.0001 | -1.22 | 0.54 | .024 |
| Adj. R^2^ | .069 |  | <.05 | .022 |  | <.05 |
| Note. NI = non-involved comparison group. ^A^dummy coded bullying victimization groups. ^B^dichotomized depression symptoms at T2 with 0=non-clinical and 1=sub-or clinical symptom levels. The most relevant (based on hypothesis testing) significant effects, based on an ⍺ of .05, are highlighted in bold. | | | | | | |

**Appendix S6**

**Appendix S7**

**Analyses with Self- and Peer-reported Bully-victim and Bully Data**

**Depression Symptoms per Bullying Victimization Group**

Overall, when simply examining the distribution of having depression symptoms by bullying victimization nomination using a crosstab, bully-victims had a .032 and bullies a .043 higher probability for depression symptoms compared with the non-involved group.

**Associations Between Self-reported Bullying Victimization, Hostility, Assertiveness, and Depression Symptoms**

When we continued to examine whether hostility and assertiveness were justified to be tested as mediators of the bullying-depression relation, we found an overall statistically significant group difference regarding hostility (*F*(3, 1024.8) = 23.04, *p* < .001) and assertiveness (*F*(3, 1004.0) = 6.94, *p* < .001), when accounting for the effect of the covariates. Post-hoc regression analyses (see Table 3 in main manuscript) showed victims, bully-victims, and bullies all having significantly more hostility than the non-involved group.

**Additional Mediation Results for Bully-victims and Bullies based on Imputed Data**

For an overview of test statistics for all mediation analyses using imputed data, see supplementary Table S4 in Appendix S8.

**Analyses with Self-reported Bullying Data.**

**Mediation of Hostility and Assertiveness on the Bully-victim-Depression Relationship**

For hostility, the indirect effect of the bully-victim dummy was significant (*p*< .0001) and positive (*a*b*= .030). Relative to the total effect (.013), the estimated proportion of the effect of being a bully-victim compared to a non-involved individual on depression symptoms due to hostility was 260%. The indirect effect for assertiveness was not significant (*p*= .170).

**Mediation of Hostility and Assertiveness on the Bully-Depression Relationship**

For hostility, the indirect effect of the bully dummy was significant (*p*< .0001) and positive (*a*b*= .026). Relative to the total effect (.060), the estimated proportion of the effect of being a bully compared to a non-involved individual on depression symptoms due to hostility was 46.3%. This means that almost half of the total effect was due to hostility, which is a moderate effect. The indirect effect for assertiveness was not significant (*p*= .077).

**Analyses with Peer-Reported Data**.

**Mediation of Hostility and Assertiveness on the Peer-reported Bully-victim-Depression Relationship**

Neither the indirect effect for hostility (*p*= .125) nor for assertiveness (*p*= .742) was significant.

**Mediation of Hostility and Assertiveness on the Peer-reported Bully-Depression Relationship**

Neither the indirect effect for hostility (*p*= .458) nor for assertiveness (*p*= .143) was significant.

**Discussion of Additional Analyses for Bully-victims and Bullies Based on Imputed Data**

Results of mediation analyses for individuals with a bully-victim or a bully nomination were very similar to our findings for the victim group (discussed in the main manuscript). We found that hostility but not assertiveness significantly explained the increased depression risk for both self-reported bully-victims and bullies, and that neither of the two interpersonal traits could explain the relation between peer-reported bullying nomination and depression symptoms. This suggests that our mediation model helped explain the risk for depression symptoms of people with any type of self-reported involvement in bullying victimization, whether that is as a victim, bully, or both. In fact, hostility was the strongest predictor of bully-victims’ depression risk, and a stronger predictor of bullies’ risk for depression symptoms as compared to victims’ risk for depression. This is in line with our descriptive data in Table 1 showing higher mean hostility scores for both pure bullies and bully-victims as compared to pure victims, and with previous research on aggression and bullying (e.g., Salmivalli & Nieminen, 2002). Additionally, more intense hostile behavior, as in bullies and bully-victims, also pulls for more intense hostile responses (Orford, 1986) and interpersonal conflict. This can explain why hostility was a stronger predictor of depression for both bullies and bully-victims than for victims.

**Appendix S8**

| Supplementary Table S4. | | | | | | | | |
| --- | --- | --- | --- | --- | --- | --- | --- | --- |
| *Estimated mediation effects of hostility and assertiveness on probability for depression symptoms per bullying victimization group (dummy-coded with non-involved (NI) as reference group) based on* ***imputed data****.* | | | | | | | | |
|  | Self-report | | | | Peer-report | | | |
|  | Hostility | | Assertiveness | | Hostility | | Assertiveness | |
|  | Estimate | *p* | Estimate | *p* | Estimate | *p* | Estimate | *p* |
|  |  |  |  |  |  |  |  |  |
| Bully-victim |  |  |  |  |  |  |  |  |
| Indirect effect (a*b) | **.030** | **<.0001** | .003 | .170 | .016 | .125 | .001 | .742 |
| Averaged direct effect | -.017 | .556 | -.016 | .568 | -.032 | .488 | -.030 | .489 |
| Total effect | .013 | .738 | -.013 | .639 | -.017 | .684 | -.029 | .505 |
| Proportion mediated | 2.611 | .738 | -.216 | .705 | -.763 | .747 | -.053 | .939 |
|  |  |  |  |  |  |  |  |  |
| Bullies |  |  |  |  |  |  |  |  |
| Indirect effect (a*b) | **.026** | **<.0001** | -.003 | .077 | .007 | .458 | -.007 | .143 |
| Averaged direct effect | .033 | .218 | .031 | .218 | .011 | .872 | .010 | .872 |
| Total effect | .060 | .027 | .028 | .268 | .018 | .779 | .004 | .984 |
| Proportion mediated | .463 | .027 | -.152 | .330 | -1.480 | .804 | .273 | .989 |
| *Note*. Significant mediation effects at an alpha of .05 are highlighted in bold. | | | | | | | | |

**Appendix S9**

**All Mediation Results Based on Observed Data**

For an overview of test statistics for all mediation analyses using observed data, see supplementary Table S2.

**Mediation of Hostility and Assertiveness on the Victim-Depression Relationship**

For hostility, the indirect effect of the victim dummy was significant (*p*< .0001) and positive (*a*b*= .026). Relative to the total effect (.060), the estimated proportion of the effect of being a victim compared to a non-involved individual on depression symptoms due to hostility was 46.0%. This means that almost half of the total effect was due to hostility, which is a moderate effect. The indirect effect for assertiveness was not significant (*p*= .204).

**Mediation of Hostility and Assertiveness on the Bully-victim-Depression Relationship**

For hostility, the indirect effect of the bully-victim dummy was significant (*p*< .0001) and positive (*a*b*= .043). Relative to the total effect (.052), the estimated proportion of the effect of being a bully-victim compared to a non-involved individual on depression symptoms due to hostility was 82.6%. This means that a large part of the total effect was due to hostility. The indirect effect for assertiveness was not significant (*p*= .199).

**Mediation of Hostility and Assertiveness on the Bully-Depression Relationship**

For hostility, the indirect effect of the bully dummy was significant (*p*= .012) and positive (*a*b*= .022). Relative to the total effect (.062), the estimated proportion of the effect of being a bully compared to a non-involved individual on depression symptoms due to hostility was 36.3%. This means that more than a third of the total effect was due to hostility, which is a moderate effect. The indirect effect for assertiveness was not significant (*p*= .504).

**Analyses with Peer-reported Data**.

**Mediation of Hostility and Assertiveness on the Peer-reported Victim-Depression Relationship**

Neither the indirect effect for hostility (*p*= .148) nor for assertiveness (*p*= .232) was significant.

**Mediation of Hostility and Assertiveness on the Peer-reported Bully-victim-Depression Relationship**

Neither the indirect effect for hostility (*p*= .116) nor for assertiveness (*p*= .566) was significant.

**Mediation of Hostility and Assertiveness on the Peer-reported Bully-Depression Relationship**

Neither the indirect effect for hostility (*p*= .388) nor for assertiveness (*p*= .252) was significant.

Regarding the significance level and direction of effects, the mediation results based on the observed and imputed data were very comparable. Based on the imputed data, we found a significant victim*assertiveness interaction using the imputed data but not the observed data.

Comparing the strengths of the mediated proportions for the significant hostility mediations, there were some differences between the observed and imputed data. In both the observed and imputed data, bully-victims had the strongest mediation effect. In the imputed data, bullies had the second strongest mediation effect, whereas in the observed data, victims had the second strongest mediation effect.

| Supplementary Table S5. | | | | | | | | |
| --- | --- | --- | --- | --- | --- | --- | --- | --- |
| *Estimated mediation effects of hostility and assertiveness on probability for depression symptoms per bullying victimization group (dummy-coded with non-involved (NI) as reference group) based on* ***observed data*** | | | | | | | | |
|  | Self-report | | | | Peer-report | | | |
|  | Hostility | | Assertiveness | | Hostility | | Assertiveness | |
|  | Estimate | *p* | Estimate | *p* | Estimate | *p* | Estimate | *p* |
| Victims |  |  |  |  |  |  |  |  |
| Indirect effect (a*b) | **.026** | **<.0001** | .004 | .204 | .012 | .148 | .011 | .232 |
| Averaged direct effect | .034 | .216 | .048 | .168 | .033 | .454 | -.072 | .262 |
| Total effect | .060 | .028 | .052 | .122 | .046 | .354 | -.061 | .368 |
| Proportion mediated | .460 | .028 | .081 | .298 | .272 | .374 | -.172 | .544 |
|  |  |  |  |  |  |  |  |  |
| Bully-victim |  |  |  |  |  |  |  |  |
| Indirect effect (a*b) | **.043** | **<.0001** | .003 | .199 | .042 | .116 | .001 | .566 |
| Averaged direct effect | .009 | .866 | -.015 | .587 | -.004 | .868 | -.029 | .332 |
| Total effect | .052 | .414 | -.012 | .657 | .038 | .852 | -.028 | .342 |
| Proportion mediated | .826 | .414 | -.228 | .721 | 1.092 | .824 | -.041 | .644 |
|  |  |  |  |  |  |  |  |  |
| Bullies |  |  |  |  |  |  |  |  |
| Indirect effect (a*b) | **.022** | **.012** | -.002 | .504 | .011 | .388 | -.012 | .252 |
| Averaged direct effect | .039 | .366 | .038 | .366 | -.008 | .860 | -.007 | .860 |
| Total effect | .062 | .202 | .036 | .400 | .018 | .980 | -.019 | .720 |
| Proportion mediated | .363 | .206 | -.060 | .772 | 3.605 | .944 | .615 | .796 |
| *Note*. Significant mediation effects at an alpha of .05 are highlighted in bold. | | | | | | | | |

**Appendix S10**

References

Bouman, T., van der Meulen, M., Goossens, F. A., Olthof, T., Vermande, M. M., & Aleva, E. A. (2012). Peer and self-reports of victimization and bullying: Their differential association with internalizing problems and social adjustment. *Journal of school psychology*, *50*(6), 759-774.

Casper, D. M., & Card, N. A. (2017). Overt and relational victimization: A meta‐analytic review of their overlap and associations with social–psychological adjustment. *Child Development*, *88*(2), 466-483. <https://doi.org/10.1111/cdev.12621>

Everaert, J., Podina, I. R., & Koster, E. H. (2017). A comprehensive meta-analysis of interpretation biases in depression. *Clinical psychology review*, *58*, 33-48.

Hayes, A. F. (2012). PROCESS: A versatile computational tool for observed variable mediation, moderation, and conditional process modeling.

Hwang, S., Kim, Y. S., Koh, Y.-J., Bishop, S., & Leventhal, B. L. (2017). Discrepancy in perception of bullying experiences and later internalizing and externalizing behavior: A prospective study. *Aggressive Behavior*, *43*(5), 493-502. <https://doi.org/10.1002/ab.21707>

Imai, K., Keele, L., & Yamamoto, T. (2010). Identification, inference and sensitivity analysis for causal mediation effects. *Statistical science*, *25*(1), 51-71.

MacKinnon, D. P., Valente, M. J., & Gonzalez, O. (2020). The correspondence between causal and traditional mediation analysis: The link is the mediator by treatment interaction. *Prevention Science*, *21*, 147-157.

Orford, J. (1986). The rules of interpersonal complementarity: Does hostility beget hostility and dominance, submission? *Psychological Review*, *93*(3), 365-377. <https://doi.org/10.1037/0033-295X.93.3.365>

Perren, S., Ettekal, I., & Ladd, G. (2013). The impact of peer victimization on later maladjustment: Mediating and moderating effects of hostile and self‐blaming attributions. *Journal of Child Psychology and Psychiatry*, *54*(1), 46-55.

Pouwels, J. L., Lansu, T. A., & Cillessen, A. H. (2016). Peer victimization in adolescence: Concordance between measures and associations with global and daily internalizing problems. *Journal of adolescence*, *53*, 195-206.

Salmivalli, C., & Nieminen, E. (2002). Proactive and reactive aggression among school bullies, victims, and bully‐victims. *Aggressive Behavior: Official Journal of the International Society for Research on Aggression*, *28*(1), 30-44.

Scholte, R. H. J., Burk, W. J., & Overbeek, G. (2013). Divergence in Self- and Peer-Reported Victimization and its Association to Concurrent and Prospective Adjustment. *Journal of Youth & Adolescence*, *42*(12), 1789-1800. <https://doi.org/10.1007/s10964-012-9896-y>

Tingley, D., Yamamoto, T., Hirose, K., Keele, L., & Imai, K. (2014). Mediation: R package for causal mediation analysis.

Valeri, L., & VanderWeele, T. J. (2013). Mediation analysis allowing for exposure–mediator interactions and causal interpretation: theoretical assumptions and implementation with SAS and SPSS macros. *Psychological methods*, *18*(2), 137.

Zimmer‐Gembeck, M. J. (2016). Peer rejection, victimization, and relational self‐system processes in adolescence: Toward a transactional model of stress, coping, and developing sensitivities. *Child Development Perspectives*, *10*(2), 122-127.
